# Stimulus Generalization in Mice during Pavlovian Eyeblink Conditioning

**DOI:** 10.1523/ENEURO.0400-21.2022

**Published:** 2022-03-21

**Authors:** F. R. Fiocchi, S. Dijkhuizen, S. K. E. Koekkoek, C. I. De Zeeuw, H. J. Boele

**Affiliations:** 1 Department of Neuroscience, Erasmus MC Rotterdam, Rotterdam 3015 GD, The Netherlands; 2 Royal Dutch Academy of Arts & Science (KNAW), Netherlands Institute for Neuroscience, Amsterdam 1105 BA, The Netherlands; 3 Princeton Neuroscience Institute, Princeton, NJ 08540

**Keywords:** auditory, cerebellum, eyeblink conditioning, motor learning, sensory system

## Abstract

Here, we investigate stimulus generalization in a cerebellar learning paradigm, called eyeblink conditioning. Mice were conditioned to close their eyes in response to a 10-kHz tone by repeatedly pairing this tone with an air puff to the eye 250 ms after tone onset. After 10 consecutive days of training, when mice showed reliable conditioned eyelid responses to the 10-kHz tone, we started to expose them to tones with other frequencies, ranging from 2 to 20 kHz. We found that mice had a strong generalization gradient, whereby the probability and amplitude of conditioned eyelid responses gradually decreases depending on the dissimilarity with the 10-kHz tone. Tones with frequencies closest to 10 kHz evoked the most and largest conditioned eyelid responses and each step away from the 10-kHz tone resulted in fewer and smaller conditioned responses (CRs). In addition, we found that tones with lower frequencies resulted in CRs that peaked earlier after tone onset compared with those to tones with higher frequencies. Together, our data show prominent generalization patterns in cerebellar learning. Since the known function of cerebellum is rapidly expanding from pure motor control to domains that include cognition, reward-learning, fear-learning, social function, and even addiction, our data imply generalization controlled by cerebellum in all these domains.

## Significance Statement

Conditioned stimuli that resemble each other will evoke a rather similar behavioral response. Here, we investigate this phenomenon of stimulus generalization using Pavlovian eyeblink conditioning to probe cerebellar function. Cerebellum is a brain structure whose known function is rapidly expanding from pure motor control to domains that include cognition, reward-learning, fear-learning, social function, and even addiction. Since we found a strong generalization of the auditory stimulus in eyeblink conditioning, our data imply an important role for generalization in motor and nonmotor domains.

## Introduction

Given the advance of transgenics and optogenetics (Deisseroth, 2011; Boyden, 2015; Navabpour et al., 2020), mice have become an increasingly popular animal model model to study mechanisms underlying cerebellar learning (Alba, 1994; Heiney et al., 2014; Kloth et al., 2015; Gao et al., 2016; Albergaria et al., 2018; Zhang et al., 2019). This also holds for Pavlovian eyeblink conditioning, a behavioral test that allows for wide variations in parameter space, including those of onset, duration and intensity of both conditional stimulus (CS) and unconditional stimulus (US; Alba, 1994; Chettih et al., 2011). Accordingly, experimental procedures for eyeblink conditioning in mice have been optimized over the last few years, evolving from Electromyography (EMG) and Magnetic Distance Measurement Technique (MDMT) to less invasive high-speed video recordings of the eyelids while the mouse is walking on a treadmill system (Koekkoek et al., 2002; Heiney et al., 2014; De Zeeuw and Ten Brinke, 2015; Albergaria et al., 2018). Even so, some basic concepts of eyeblink conditioning, which have been studied quite extensively in humans and rabbits, have not yet been studied well in mice.

One of these concepts is called “stimulus generalization.” Stimulus generalization is the phenomenon whereby a certain behavioral response is elicited by a stimulus other than the one that initially led to the acquisition of this specific response (Lashley and Wade, 1946; Razran, 1949; Pavlov, 2010). Stimulus generalization plays a crucial role in our daily life behavior. Think for instance about how we, as a pedestrian, will immediately respond to the sound of *any* car horn when we are about to cross a road. Indeed in neuroscience, stimulus generalization has probably been most extensively studied in the context of Pavlovian fear conditioning (Hovland, 1937; Bang et al., 2008; Lissek et al., 2008; Meulders and Vlaeyen, 2013; Dymond et al., 2015), showing that emotional and fight-or-flight responses can be easily triggered by stimuli other than the one used during acquisition training.

In the current study, we employ Pavlovian eyeblink conditioning in mice to study the stimulus generalization of their responses that reflect motor memories. In the paradigm used in this study, mice were first trained to close their eyes in response to a tone with a frequency of 10 kHz, by repeatedly pairing this 10-kHz tone (CS) with a mild air puff to the eye (US). After 10 consecutive daily training sessions, which is sufficient for most mice to reliably show eyelid conditioned responses (CRs) to the 10-kHz tone, we suddenly introduced alternative tones with frequencies ranging between 2 and 8 and 12 and 20 kHz. In contrast to the 10-kHz tone, these tones were never reinforced with the aversive air puff. In line with previous work (Dymond et al., 2015), we will use in this paper the term conditional stimulus or CS to refer to the 10-kHz tone that was reinforced with the air puff US, and the term generalization stimulus (GS) for the other tones, varying between 2 and 8 and 12 and 20 kHz, that were *never* reinforced with the air puff US. Thus, we set out to investigate to what extent eyeblink CRs are elicited by these GSs in mice that do show reliable CRs to the CS.

The advantage of eyeblink conditioning is that it does not only permit robust variations in the stimulus parameters, but also that it reveals the CR probability as well as quantification of the size (or amplitude) and timing of the CRs. Eyeblink CRs are not simple reflexive blinks in response to the tone, but precisely timed responses, the amplitude of which increase gradually over the course of training (Boele et al., 2016). The adaptive timing of eyeblink CRs depends on the interval between CS and US onset: after conditioning the eye will be maximally closed just before the moment that the air puff (i.e., US) will be delivered. Thus, in this study we quantified CR probability, CR amplitude, and CR timing as a function of tone frequency, allowing us to investigate to what extent these different parameters are subject to the stimulus generalization principle.

## Materials and Methods

### Subjects

We used 14 wild-type C57Bl/6 mice, seven of which were male and seven were female. Mice were between 11 and 16 weeks old at the start of the experiment. All mice were housed individually during the experiment with food and water *ad libitum* in a 12/12 h light/dark cycle. Experiments were performed during the light phase. All experiments were performed in accordance with the European Communities Council Directive. Protocols were reviewed and approved by the Erasmus Laboratory Animal Science Center (work protocol no. 15-273-138; project license no. AVD101002015273)

### Auditory brainstem responses (ABRs)

Since C57Bl/6 mice are prone for developing hearing problems, we recorded the ABRs before the start of eyeblink conditioning training to obtain hearing level thresholds. Mice were anesthetized with a ketamine/xylazine mixture at a dose of 100/10 mg/kg body weight, administered intraperitoneally (ketamine: Alfasan; xylazine: Sedazine, AST Farma). After this, they were placed in a sound-attenuated and light-attenuated box with the ears at a distance of 4 cm from a frontally placed loudspeaker. Needle electrodes were positioned subdermal at the base of both pinnae, the external part of the ear. The reference electrode was placed at the vertex, the upper surface of the head, and a ground electrode on the lower back. Stimuli were generated and presented by a RZb Multi I/O Processor (TuckerDavis Technologies) and BioSigRZ software. Responses were recorded using Medusa DA4PA, 4-dh Preamp device. Responses with amplitudes larger than 30 μV were considered as artefacts and therefore excluded from further analysis. Hearing level thresholds were measured at 4, 8, 16, and 32 kHz (Willott, 2006). Thresholds were defined as the lowest sound pressure level (SPL) at which a reproducible response (i.e., peak in the ABR trace) was still detectable. Since our main aim was to establish that mice could detect the tones used in our behavioral training paradigm and not to establish definite age and mouse species dependent absolute hearing thresholds, we performed our ABR recordings under anesthesia since it is technically less complicated. One should keep in mind, however, that ABR responses under ketamine/xylazine anesthesia, although it is a standard procedure in mice (Huang et al., 1995; Willott, 2006; Ingham et al., 2011), are generally weaker compared with those recorded in awake animals (van Looij et al., 2004). After the ABR recordings, which took ∼20–30 min per animal, mice were injected with atipamezole (Antisedan, Orion Pharam; 10 mg/kg body weight, i.p.) for the reversal of xylazine.

### Surgery

After ABR recordings, mice had 2 d of recovery before they underwent surgery. Mice were anesthetized with 2% isoflurane (vaporizer for Isoflurane Anesthetic Model100 Vaporizer, Forane, Surgivet) and body temperature was kept constant at ∼37°C (DC Temperature controller, FHC). After fixation in a standard mouse stereotaxic alignment system (Stoelting) and under sterile conditions, the scalp was incised (∼10 mm) to expose the skull. Membranous tissue was cleared and the bone was prepared with Optibond FL (All-in-one bonding agent Kerr). A small brass pedestal with a square magnet on top was attached to the skull with a dental composite (Charisma, Mitsui Chemical Group), using an xyz manipulator, allowing for fixation to a head bar at right angles during experiments. After surgery mice recovered under a heating lamp for at least 20 min, until they were fully awake. They were given postoperative analgesic (Rimadyl Cattle, Cappelle a/d.) on the following day. Mice had 3 d to fully recover, before eyeblink conditioning habituation training started.

### Eyeblink conditioning, apparatus

**Figure 1.**
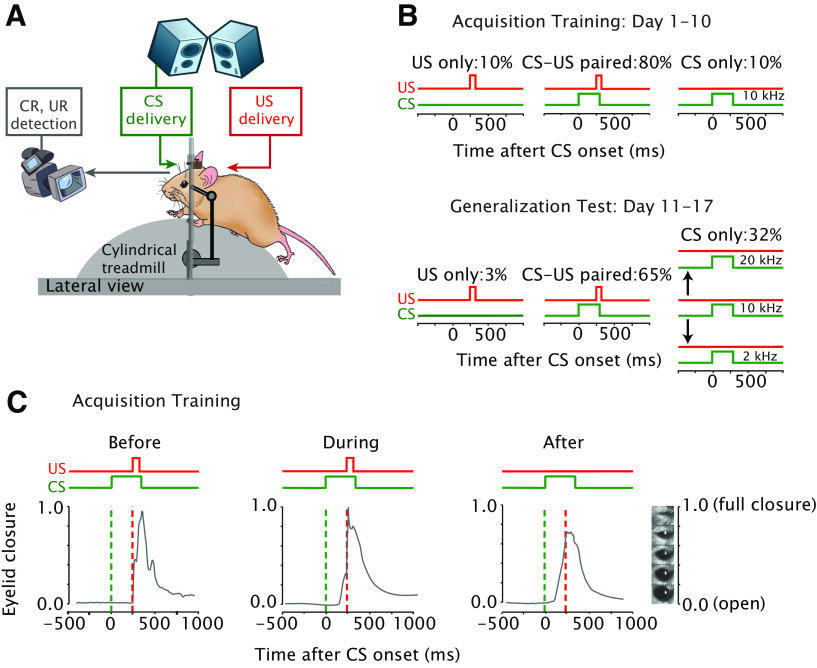
Eyeblink conditioning setup and experimental design. ***A***, Mice were placed in a light-isolating and sound-isolating chamber on a foam cylindrical treadmill that allowed them to walk freely with their heads fixed at a horizontal bar. The unconditioned stimulus, US (in red) consisted of a weak air puff to the left eye and the conditioned stimulus, CS (in green) consisted of a 10-kHz tone. Speakers were placed on both upper front corners of the chamber. Eyelid movements were recorded using a high-speed video camera system (300 fps). ***B***, Schematics of eyelid conditioning acquisition training and generalization test protocols. For each protocol the duration and the ratio of different trial types is presented at the top of the corresponding illustration. ***C***, Example eyeblink traces before, during, and after eyeblink conditioning. The CS (green) and US (red) onset and duration are shown at the top of each panel. Over the course of acquisition training, mice learn to close their eyes in response to the CS, which are called conditioned responses (CRs).

All behavioral experiments were conducted in custom built sound-attenuating and light-attenuating boxes. Mice were placed head-fixed on top of a cylindrical treadmill on which they were allowed to walk freely (Fig. 1*A*; Heiney et al., 2014; Boele et al., 2018). The treadmill consisted of a foam roller (diameter, ±15 cm; width, ±12 cm; Exervo, TeraNova EVA) with a horizontal metal rod through the axis that was connected with a ball bearing construction to two solid vertical metal poles. A horizontal messing bar was fixated to the same vertical poles at 3–5 cm above the treadmill. Mice were head-fixed to the bar with the use of a screw, allowing the magnet on top of the pedestal to perfectly dovetail another magnet with opposite polarity in the middle of the horizontal messing bar in the exact point of fixation, thereby ensuring easy fixation and perfect head stability (Fig. 1*A*; Chettih et al., 2011; Heiney et al., 2014; Boele et al., 2018). The CS was a 280-ms tone with a frequency of 10 kHz with a 25-ms ramp/decay time. The US consisted of a 30-ms duration mild corneal air puff, which was controlled by a VHS P/P solenoid valve (Lohm rate, 4750 Lohms; Internal volume, 30 μl, The Lee Company) and delivered via a 27.5-mm gauge needle that was perpendicularly positioned at ∼5 mm from the center of the left cornea. The back pressure on the solenoid valve was set at 30 psi. We used an interstimulus interval of 250 ms and an intertrial interval of 8–12 s. Eyelid movements were recorded using a high-speed video camera (333 fps, Basler a cA640-750u m ID: 106748-15). Stimulus control and data acquisition were done with National Instruments hardware. All experiments were performed at approximately the same time of the day by the same experimenter.

### Eyeblink conditioning, habituation to eyeblink conditioning apparatus

Mice were head-fixed on the head bar and allowed to walk on the treadmill for 20–30 min/d for 2 d without any stimuli to get them acquainted with the eyeblink set-up.

### Eyeblink conditioning, baseline measurements to find the proper tone threshold for each animal

**Figure 2.**
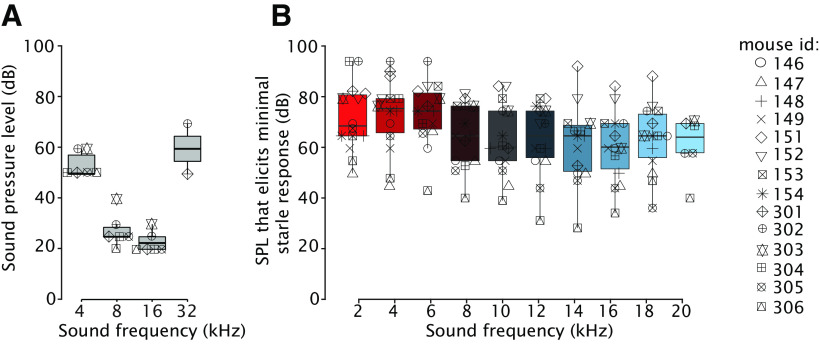
Mouse ABRs and auditory eyelid startle responses to a range of sound frequencies and intensities. ***A***, ABRs were measured at 4, 8, 16, and 32 kHz. Mice were most sensitive to sounds in the range of 8 and 16 kHz showing normal auditory thresholds. Each symbol represents one mouse. For the boxplot, the thick horizontal line is showing the median, the top edge of each box indicates the 25th percentile, bottom edge the 75th percentile, whisker lines extending above and below each box indicate the range of observations. ***B***, The SPLs that elicits minimal auditory eyelid startle response for different sound frequencies. This value was determined for each mouse carefully during 10 baseline sessions and used as CS or GS.

**Figure 3.**
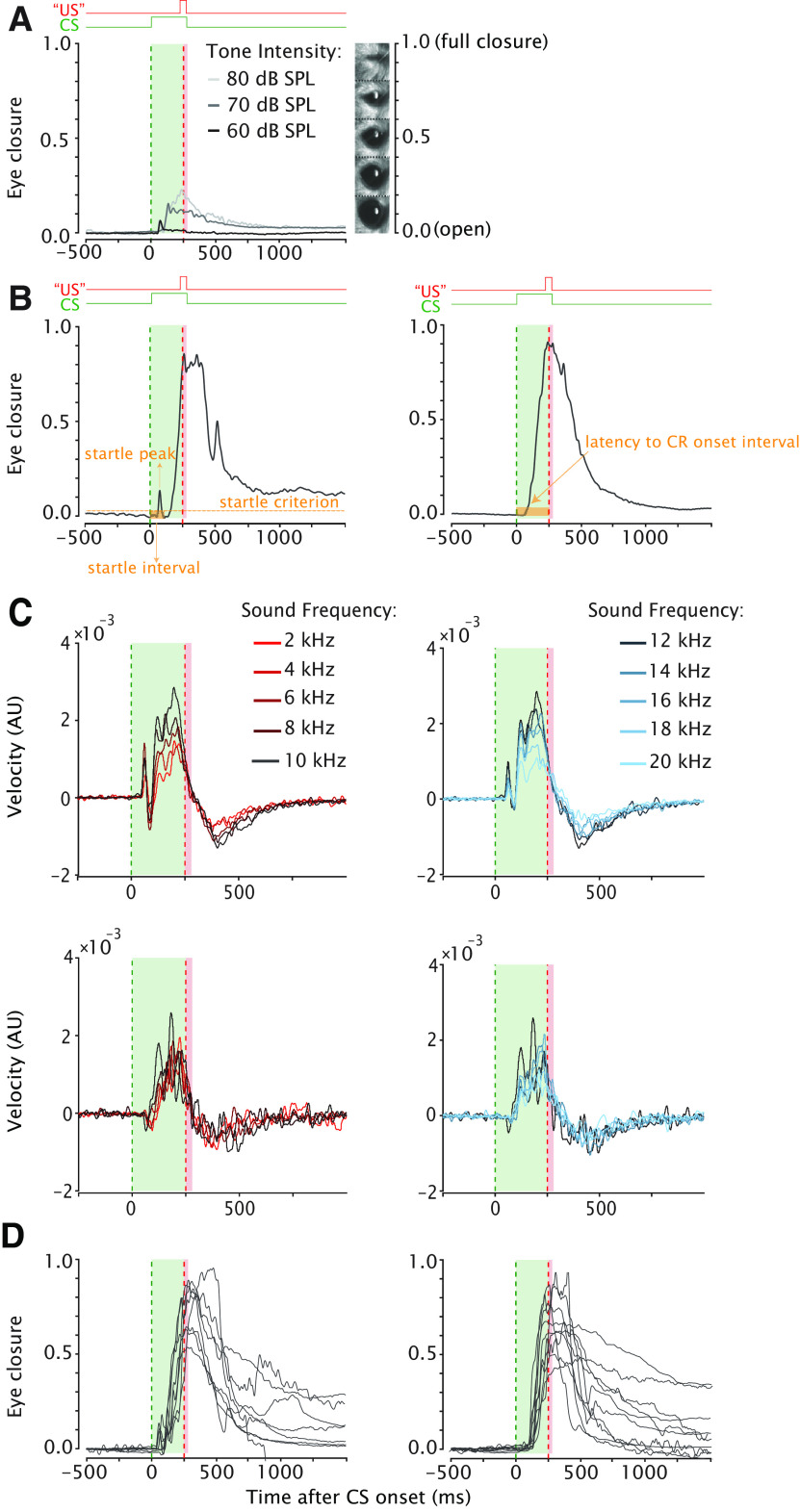
Mouse eyelid startle responses obscure CR onset. ***A***, Example eyelid responses to a 10-kHz tone in a naive unconditioned mouse presented at three different sound intensities: 60, 70, and 80 decibels (dB). In this example the 60-dB tone elicits just an α startle response, the 70-dB tone an α and β startle response, and the 80 an α and β startle and even a response that resembles a CR. For this mouse, a tone with a SPL of 60 dB would be a proper CS for training. ***B***, Example eyelid responses after training taken from the same animal. In the left panel, the α startle response obscures the CR onset. In the right panel, there is no startle response and consequently the latency to CR onset can be detected reliably. ***C***, Separation of startle and nonstartle trials was achieved by taking the first derivative of the eyelid position signal. In this velocity signal, the presence of a peak immediately after CS onset was the discriminator between startle (top panels) and nonstartle trials (bottom panels). Latency to CR onset was determined in nonstartle trials. For all other outcome measures, startle and nonstartle trials were combined. Similar to Figure 2, the blue gradient indicates GSs with frequencies higher than the 10-kHz CS and red gradient indicates GSs with frequencies lower than the 10-kHz CS. Each line is the averaged velocity signal of the eyeblink trace for one GS or CS frequency. For all panels, the green dashed line indicates CS onset, the red dashed line indicates expected US onset. The light green and red shadings indicate CS and US duration, respectively. Eyelid is fully open at 0 and fully closed at 1. The US is omitted in the CS-only trials. CS (tone), US (eye puff). ***D***, Eyelid responses separated by the presence of an α startle response. Left panel shows eyelid traces with a startle response, right panel shows traces without a startle response.

After the 2 d of habituation, we measured the sensitivity of each mouse for the tone CS (10 kHz) and tone GSs (ranging from 2 to 8 and 12 to 20 kHz in steps of 2 kHz). Since the responsiveness of an individual mouse to auditory stimuli can slightly vary from day to day, and since we were testing 10 different tone frequencies and did not want to present too many trials during a baseline session, we repeated this measurement for 10 consecutive days for each animal (30 min each day), until we found for each animal the proper SPLs (SPL in dB) for each tone frequency (Figs. 2*B*, 3*A*). Each baseline session consisted of two blocks of 10 tone-only trials and 1 US-only trial each, and thus had two tone-only trials of each tone frequency for each session.

**Table 1:** Eyeblink conditioning outcome measures during acquisition training (sessions 1–10)

Session	CR percentage	CR amp-all trials	CR amp-CR only trials	Latency to CR peak	Latency to CR onset
1	14 (±8)	0.11 (±0.05)	0.19 (±0.06)	329.7 (±51.1)	130.7 (±276.3)
2	33 (±12)	0.23 (±0.10)	0.30 (±0.12)	306.7 (±31.6)	124.9 (±41.5)
3	37 (±16)	0.27 (±0.16)	0.39 (±0.19)	314.3 (±28.4)	116.5 (±31.8)
4	38 (±13)	0.30 (±0.19)	0.44 (±0.21)	302.6 (±22.7)	137.8 (±56.7)
5	42 (±15)	0.37 (±0.15)	0.46 (±0.20)	303.4 (±28.0)	139.9 (±44.8)
6	50 (±15)	0.40 (±0.15)	0.53 (±0.17)	313.8 (±30.9)	114.0 (±23.7)
7	56 (±16)	0.33 (±0.12)	0.44 (±0.13)	313.5 (±28.2)	145.7 (±61.8)
8	63 (±17)	0.48 (±0.15)	0.56 (±0.14)	310.0 (±24.2)	122.1 (±95.8)
9	67 (±14)	0.48 (±0.11)	0.59 (±0.10)	312.5 (±24.6)	119.2 (±37.1)
10	67 (±14)	0.45 (±0.11)	0.56 (±0.10)	313.5 (±29.2)	151.5 (±19.5)
ANOVA on LME	*F*_(9,90)_ = 10.85, *p* < 0.0001	*F*_(9,2000)_ = 16.56, *p* < 0.0001	*F*_(9,931)_ = 8.14, *p* < 0.0001	*F*_(9,931)_ = 0.62, *p* = 0.77	*F*_(9,90)_ = 1.47, *p* = 0.17

All values represent mean ± 95% CI. The ANOVA on linear mixed-effect (LME) model shows the main effect of session. CR, conditioned response.

**Table 2:** Eyeblink conditioning outcome measures during generalization test sessions (sessions 11–17)

Tone freq.	CR percentage	CR amp-all trials	CR amp-CR only trials	Latency to CR peak	Latency to CR onset
2 kHz	38 (±6)	0.20 (±0.04)	0.42 (±0.05)	310.0 (±13.9)	162.5 (±15.0)
4 kHz	43 (±7)	0.28 (±0.06)	0.49 (±0.07)	323.9 (±15.7)	147.8 (±17.9)
6 kHz	50 (±6)	0.34 (±0.05)	0.54 (±0.05)	311.0 (±9.10)	158.1 (±21.4)
8 kHz	58 (±6)	0.42 (±0.04)	0.56 (±0.04)	315.1 (±9.79)	148.8 (±23.3)
**10 kHz**	**67 (±5)**	**0.51 (±0.04)**	**0.63 (±0.04)**	**320.1 (±8.96)**	**145.3 (±16.0)**
12 kHz	65 (±5)	0.46 (±0.04)	0.60 (±0.04)	311.5 (±9.72)	151.3 (±18.9)
14 kHz	63 (±5)	0.43 (±0.04)	0.59 (±0.04)	327.8 (±10.3)	142.6 (±13.1)
16 kHz	61 (±4)	0.40 (±0.03)	0.55 (±0.04)	324.9 (±11.8)	155.8 (±13.4)
18 kHz	55 (±6)	0.31 (±0.04)	0.47 (±0.05)	332.1 (±12.5)	141.2 (±16.0)
20 kHz	55 (±6)	0.29 (±0.04)	0.46 (±0.05)	341.3 (±13.6)	152.8 (±13.3)
ANOVA on LME	*F*_(9,726)_ = 11.99, *p* < 0.0001	*F*_(9,4849)_ = 44.34, *p* < 0.0001	*F*_(9,2692)_ = 16.70, *p* < 0.0001	*F*_(9,2692)_ = 5.56, *p* < 0.0001	*F*_(9,322)_ = 1.12, *p* = 0.34

All values represent mean ± 95% CI. The ANOVA on linear mixed-effect (LME) model shows the main effect sound frequency. *Post hoc* comparisons are shown in Figure 6 and Tables 3, 4. CR, conditioned response. Bold values represent outcome measures values in response to the trained CS of 10-kHz during stimulus generalization test.

As a behavioral readout for the tone sensitivity, we used the eyelid component of the auditory startle reflex (Boele et al., 2010). This startle response, sometimes referred to as α response, was quantified using the velocity signals (first derivative of position signal; Fig. 3*C*). An eyelid response was considered as a startle response if there was a peak in the velocity signal between 30 and 80 ms after CS onset that was larger than 3 SDs of the 500-ms baseline period and larger than an arbitrary threshold set at 0.00025 (Fig. 3*B*,*D*). We considered the potential effect of “latent inhibition,” which is the phenomenon whereby it takes longer to get conditioned to a familiar stimulus (i.e., a tone that one has heard many times) than to novel stimulus (i.e., a new tone). Therefore, all animals received the exact same number of CS-only trials during baseline, so that the novelty level of the tone was equal for all animals.

### Eyeblink conditioning, acquisition training sessions

Mice were trained for 10 consecutive days (40 min/d). Each daily session was composed of 20 blocks of 12 trials each. Each block consisted of 1 US-only trial, 10 paired (CS-US with an interstimulus interval, ISI of 250 ms) trials, and one CS-only trial (Fig. 1*B*,*C*). Trials were semi-randomly distributed, whereby a CS-only trial was always immediately preceded by at least two paired CS-US trials.

### Eyeblink conditioning, generalization test sessions

The 10 d of acquisition training were followed by seven generalization test sessions, during which the GSs were presented in addition to the CS. Each daily generalization test session was composed of eight blocks of 31 trials each, including 20 paired CS-US trials, one US-only, and 10 tone (CS or GS) only trials (Fig. 1*B*). Since the ratio of paired CS-US to CS/GS only trial changed significantly, we carefully checked day-by-day whether there was any extinction of eyeblink CRs. Since it is known that the probability of a CR is lower in a trial that is preceded by a trial wherein the tone was not reinforced (Najafi and Medina, 2020), in our experimental design a tone only trial (CS only or GS only) was always immediately preceded by two paired CS-US trials.

### Eyeblink conditioning, data analysis

Individual eyeblink traces were analyzed with a custom-written MATLAB script (R2018a, MathWorks). First, the 2000-ms eyeblink traces were imported from the MySQL database into MATLAB. The trials were aligned at zero for the 500-ms pre-CS baselines. Trials with significant activity in the 500-ms pre-CS period (more than seven times the interquartile range) were regarded as invalid and disregarded for further analysis. The eyelid signal was min-max normalized so that a fully open eye corresponded with a value of 0 and a fully closed eye with a value of 1. This normalization was achieved by aligning the 500-ms pre-CS baselines of all traces and dividing each trace by the averaged UR value that was calculated over all eyelid traces in US only trials for one session. The normalized eyelid closure amplitude was expressed as fraction eyelid closure (FEC).

In our analysis we only included CS-only trials, since these trials show the full kinetic profile of the eyelid response. In valid normalized CS-only trials, eyelid responses were considered as a CR if the maximum amplitude was larger than 0.05 in the interval between 100–500 ms after CS onset and the presence of a positive slope in the 150-ms before the time point where the US would have been delivered (US is omitted in CS-only trials). For each session for each mouse, we calculated the percentage of trials in which a CR was present, which we will refer to as “CR percentage.” In addition, we determined for each trial the maximum eyelid closure between 100 and 500 ms after CS onset, which we will refer to as “Eye closure – all trials.” Similarly, we calculated the maximum eyelid closure between 100 and 500 ms after CS onset in trials wherein a CR was present based on the criteria described above, which we will refer to as “Eye closure – CR trials.” CR adaptive timing was investigated by calculating the latency to the onset of the CR relative to CS onset, referred to as “Latency to CR onset,” and the latency to maximum eye closure relative to CS onset, referred to as “Latency to CR peak.” Latency to CR onset and latency to CR peak were only calculated in trials wherein a CR was present. Latency to CR onset was only calculated for trials wherein no α startle response was present.

Statistical analysis was done using multilevel linear mixed-effect (LME) models in R Studio (code available on request). LMEs have several major advantages over standard parametric and nonparametric tests (Aarts et al., 2014; Schielzeth et al., 2020), as they are more robust to violations of normality assumptions, which is often the case in biological data samples. Moreover, use of LME models is able to accommodate the nested structure of our data (i.e., trial nested within session, session nested within animal, animal nested within group). Finally, LME models are objectively better at handling missing data points than repeated measures ANOVA models and do not require homoscedasticity as an inherent assumption. In our LME, we used session and tone frequency as fixed effects, and mouse as a random effect. Goodness of fit model comparison was determined by evaluating log likelihood ratio, BIC, and AIC values. The distribution of residuals was inspected visually by plotting the quantiles of standard normal versus standardized residuals (i.e., Q-Q plots). Correction for multiple comparisons was achieved, using false-discovery rate (FDR). Data were considered as statistically significant if the corrected *p*-value was <0.05.

## Results

We used eyeblink conditioning to test stimulus generalization in mice that were conditioned using a 10-kHz tone. Before we started the eyeblink conditioning acquisition training, we carefully measured the sensitivity of each mouse to the specific tones used during the experiment.

### Auditory brainstem and auditory startle responses

Some mouse strains, including the popular C57Bl/6 mouse strain, are susceptible to age-related hearing loss. On the other end, mice in general are anxious prey animals that are very sensitive to sounds and easily startle (Turner et al., 2005; King et al., 2015; Dent et al., 2018). To this end, we tested both ABRs and auditory startle responses in the mice before the start of the training. ABRs were measured in mice at the age of nine weeks after birth. We followed the standardized protocol during which clicks were presented at 4, 8, 16, and 32 kHz (Willott, 2006; Akil et al., 2016). At the lowest frequency of 4 kHz, a SPL of 53 dB was needed to elicit a reliable ABR peak (Fig. 2*A*). ABR peaks were elicited with the lowest SPL of 23 dB at a frequency of 16 kHz. Our results are in line with previous work testing ABRs in various mouse strains, including C57Bl/6J, at roughly the same age range (Zheng et al., 1999; Ison et al., 2007). Since we found recognizable ABR peaks in the entire range of 4–32 kHz, and since our findings are in line with these previous reports in mice (Heffner and Heffner, 2007; Reynolds et al., 2010), we conclude that hearing was intact in our animals.

Next, we established for each mouse the auditory startle response threshold. One component of the auditory startle response is a quick, partial, eyelid closure with a latency to peak of ∼50 ms. Sometimes this α startle is followed by a β startle, or short-latency response (SLR), which has a latency to peak of ∼100 ms, and these β startles can easily mask and sometimes even mimic cerebellar CRs. For this reason, we carefully determined at each tone (CS and GS) frequency for each mouse the SPL that was just sufficient to elicit a very small α startle response, but absolutely no β startle (Boele et al., 2010). The sound frequencies used ranged from 2 to 20 kHz in steps of 2 kHz; all stimuli had the same duration and ramp/decay pattern as those of the CS and GSs. Importantly, during the baseline measurement, these tones were never reinforced with an eye puff US. The baseline sessions were repeated for 10 d, each day consisting of 20 trials, which was necessary to find a proper SPL for each frequency and for each animal. To avoid the potentially differentiating effect of latent inhibition (Lubow and Moore, 1959; Lubow, 1973) between animals, all mice received the exact same amount of tone-only trials during the baseline session.

Responses to tone-only trials at different tone frequencies for the last baseline session revealed that there was considerable variation in sound sensitivity between mice (Fig. 2*B*). However, response thresholds for different frequencies within each mouse looked quite uniform (Fig. 2*B*). In line with the ABR measurements, and in agreement with previous work (Heffner and Heffner, 2007; Reynolds et al., 2010), we found that mice tended to be more sensitive, i.e., startled more easily at higher tone frequencies (16–20 kHz) than at lower frequencies (2–6 kHz). For this reason, the GS at the higher frequencies were delivered at slightly lower SPLs than those at the lower frequencies (Fig. 2*B*). Once the proper SPLs were established for each mouse for all tone frequencies, including the 10-kHz CS, these values were not changed anymore during the subsequent acquisition training sessions (days 1–10) and generalization test sessions (days 11–17).

### Eyeblink conditioning, acquisition sessions (days 1–10)

**Figure 4.**
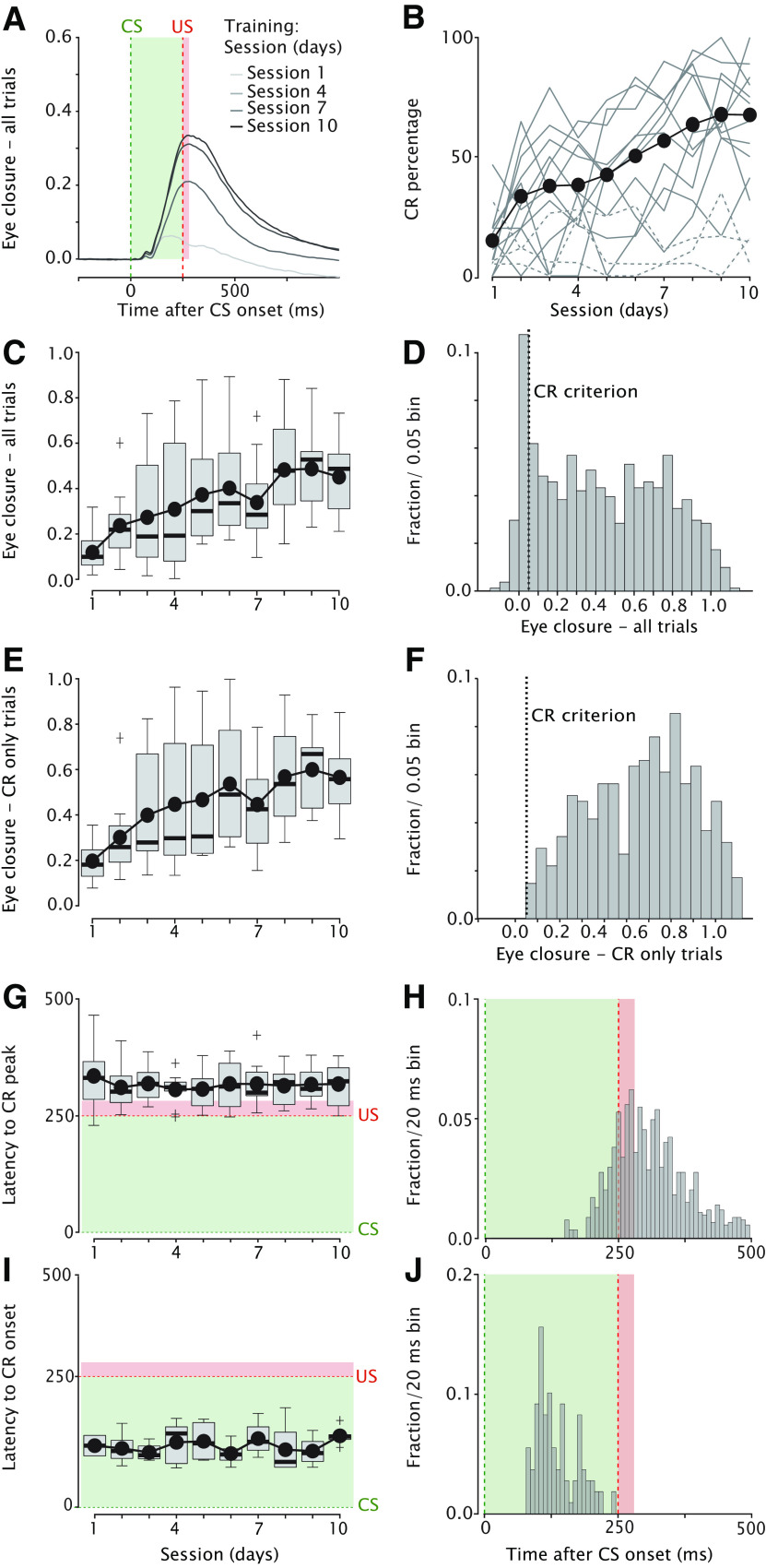
Mice acquire conditioned eyelid responses over the course of 10 consecutive training sessions. ***A***, Averaged eyeblink traces in CS-only trials during acquisition sessions 1, 4, 7, and 10 for the 11 mice that learned the task. The green dashed line indicates CS onset, the red dashed line indicates expected US onset. The light green and red shadings indicate CS and US duration, respectively. Eyelid is fully open at 0 and fully closed at 1. The US is omitted in the CS-only trials. ***B***, CR percentage as a function of acquisition training session. Each solid gray line represents a mouse that did learn the task (*n* =11), each dotted gray line represents an animal that did not learn the task, i.e., did not reach a CR percentage of >20 after 10 training days (*n* = 3). Black line with black filled dots indicates the mean of each session for the 11 animals that learn the task. ***C***, Eyelid closure amplitude over all trials plotted as a function of acquisition training session. The effect of session is statistically significant. For the boxplot, the thick horizontal line is showing the median, the top edge of each box indicates the 25th percentile, bottom edge the 75th percentile, whisker lines extending above and below each box indicate the range of observations, the plus symbols indicate outliers. The black line plot with filled black dots indicates the mean for each acquisition session. ***D***, Distribution of eyelid closure amplitude calculated over all trials (acquisition sessions 8–10 pooled). Center of mass is around 0. For calculating the Eye closure – CR only trials, in panels in ***E–J***, we used a CR criterium of 0.05 indicated with the vertical dashed line. ***E***, Similar to C but now showing eyelid closure amplitude over CR only trials plotted as a function of acquisition training session. The effect of session is statistically significant. ***F***, Similar to ***D***, but now showing the distribution of eyelid closure amplitude calculated over CR only trials (acquisition sessions 8–10 pooled). ***G***, Latency to CR peak plotted as a function of training session. The green dashed line indicates CS onset, the red dashed line indicates US onset. The light green and red shadings indicate CS and US duration, respectively. There is no statistically significant effect of session. ***H***, Distribution of latency to CR peak for all trials (acquisition sessions 1–10 pooled). Note the adaptive timing of eyeblink CRs, whereby the CR peaks around the expected US (US is omitted in CS-only trials). ***I***, Similar to ***G***, but now showing latency to CR onset plotted as a function of training session. There is no statistically significant effect of session. ***J***, Similar to ***H***, but now showing the distribution of latency to CR onset for all sessions. For complete statistics for all panels, we refer to Table 1.

Eyeblink acquisition training started the day after the last baseline session. Mice were trained for 10 consecutive days. Inspection of averaged traces showed that the size of eyelid responses to the CS gradually increased over the course of 10-d acquisition training (Fig. 4*A*). We quantified the CR percentage and found that three mice did not show a significant increase (Fig. 4*B*, gray dashed lines). These three mice were therefore disregarded in any further analysis, since our main question focuses on stimulus generalization in animals that had learned the task properly. In the remaining 11 animals, we found a statistically significant main effect of session for the average CR percentage (Fig. 4*B*; Table 1; *F*_(9,90)_ = 10.85, *p* < 0.0001, ANOVA on LME). Mice reached a stable CR percentage of ∼65–70 around day 8 that did not further increase. We did not find an effect of sex on CR probability, male and female mice showed identical learning curves (Fig. 9*A*; *F*_(9,1)_ = 0.07, *p* = 0.79, ANOVA on LME). Further quantification of the eyelid response amplitudes, revealed that there was a statistically significant main effect of session for CR amplitude calculated over all trials (Fig. 4*C*; Table 1; *F*_(9,2000)_ = 16.56, *p* < 0.0001, ANOVA on LME). Similar to CR percentage, there was no further increase after day 8. Based on the distribution of all eyelid responses (in Fig. 4*D*, sessions 8–10 pooled), we set an arbitrary cutoff at a FEC of 0.05 to distinguish between CRs (≥0.05) and non-CRs (<0.05). We calculated the CR amplitude over the CR only trials and found a similar main effect of session (Fig. 4*E*; Table 1; *F*_(9,931)_ = 8.14, *p* < 0.0001, ANOVA on LME). (Also, see the distribution of FEC calculated over CR only trials from sessions 8–10 pooled in Fig. 4*F*.)

**Table 3:** *Post hoc* comparison between the 10-kHz CS and all other GS frequencies for CR amplitude calculated over all trials and CR amplitude calculated over CR trials

CS 10 kHz vs GS	2 kHz	4 kHz	6 kHz	8 kHz	12 kHz	14 kHz	16 kHz	18 kHz	20 kHz
CR amp-all trials	*p* < 0.0001	*p* < 0.0001	*p* < 0.0001	*p* < 0.0001	*p* = 0.056	*p* < 0.0001	*p* < 0.0001	*p* < 0.0001	*p* < 0.0001
CR amp-CR only trials	*p* < 0.0001	*p* < 0.0001	*p* = 0.0004	*p* = 0.0002	*p* = 0.076	*p* = 0.021	*p* < 0.0001	*p* < 0.0001	*p* < 0.0001

All values represent FDR corrected *post hoc* comparisons using an ANOVA on linear mixed-effect (LME) model. CS, conditional stimulus; GS, generalization stimulus.

Finally, we looked in more detail at the adaptive timing of eyeblink CRs. As expected, the latency to CR peak showed a clear distribution centered around the onset of the expected US at 250 ms after CS onset (Fig. 4*G*,*H*) that remained stable over 10 acquisition sessions. We found no statistically significant effect of session for latency to CR peak (Fig. 4*G*; Table 1; *F*_(9,931)_ = 0.62, *p* = 0.77, ANOVA on LME). For the latency to CR onset, we could only use 272 out of 1018 CR trials, because of α startle response that obscured the CR onset (Fig. 3*D*). Similar to latency to CR peak, we could not find an effect of session for latency to CR onset (Fig. 4*I*,*J*; Table 1; *F*_(9,90)_ = 1.47, *p* = 0.17; ANOVA on LME). Overall, we concluded that the majority of animals showed normal eyeblink conditioning in terms of CR percentage, CR amplitude, and CR timing.

### Eyeblink conditioning, generalization test session (days 11–17)

**Figure 5.**
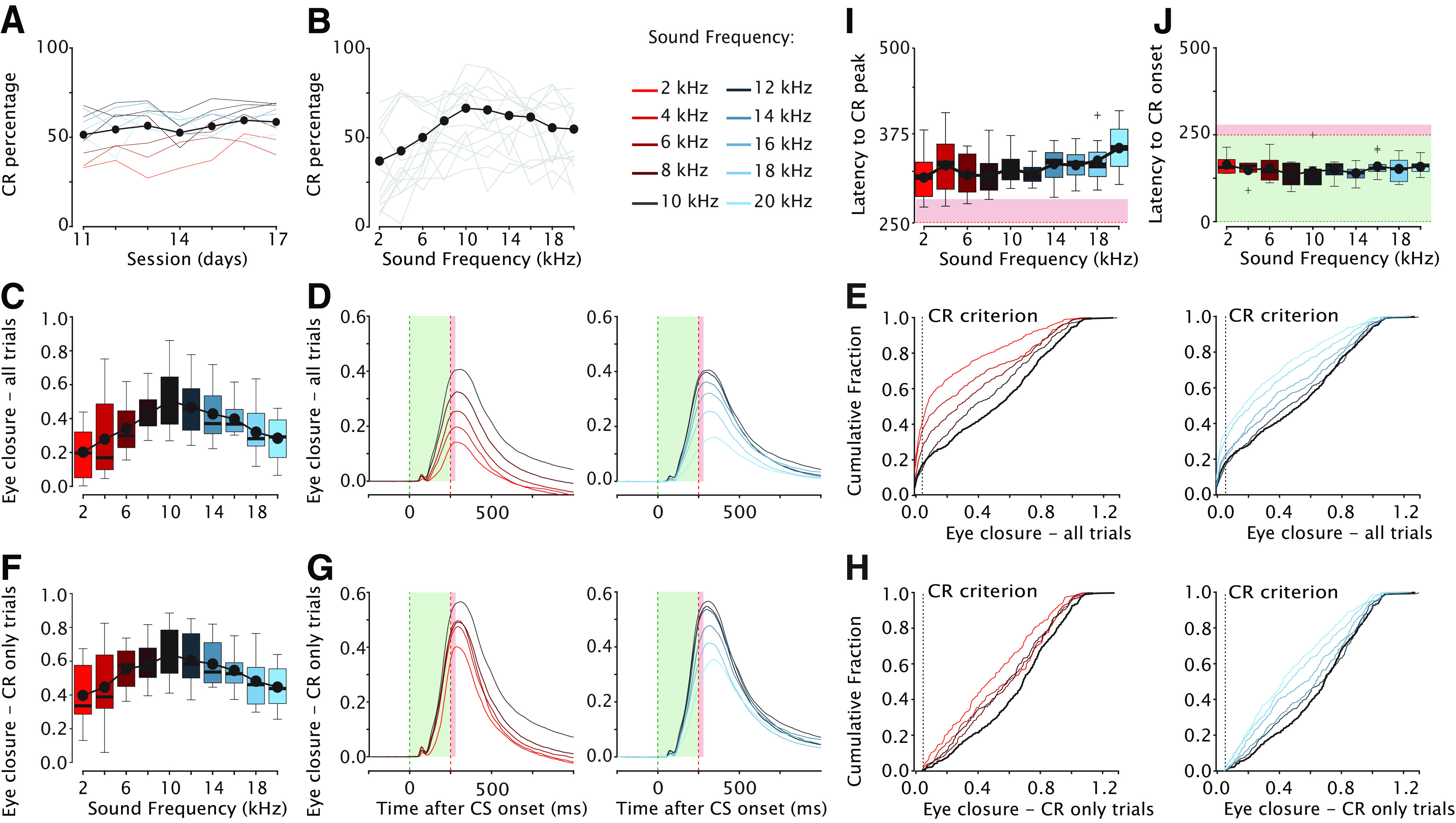
Generalization of conditioned eyelid responses in mice. ***A***, CR percentage as a function of generalization test session. Solid gray lines represent individual mice and the black line with black filled dots indicates the mean of each session. No effect was found for generalization session, indicating that there was no extinction of eyeblink CRs during the generalization test sessions. ***B***, CR percentage as a function of sound frequency. The 10-kHz tone is the CS, all other tone frequencies serve as GSs that are never reinforced with an air puff US. Solid gray lines represent individual mice, black line with black filled dots indicates the mean percentage CR for each GS. ***C***, Eyelid closure amplitude over all trials plotted as a function of sound frequency. For the boxplot, the thick horizontal line is showing the median, the top edge of each box indicates the 25th percentile, bottom edge the 75th percentile, whisker lines extending above and below each box indicate the range of observations, the plus symbols indicate outliers. The black line plot with filled black dots indicates the mean for each sound frequency used. For panels ***C–H***, the blue gradient indicates GSs with frequencies higher than the 10-kHz CS and red gradient indicates GSs with frequencies lower than the 10-kHz CS. ***D***, Averaged eyeblink traces in response to different sound frequencies. The green dashed line indicates CS onset, the red dashed line indicates expected US onset. The light green and red shadings indicate CS and US duration, respectively. Eyelid is fully open at 0 and fully closed at 1. The US is omitted in the CS-only trials. Note the symmetric generalization gradient. ***E***, Cumulative distribution function of eyelid closure calculated over all trials for the different sound frequencies. ***F***, Similar to ***C***, but now only for trials with a CR. ***G***, Similar to ***D***, but now for trials with a CR. ***H***, Similar to ***E***, but now for trials with a CR. ***I***, Effect of sound frequency on the latency to CR peak. Lower tones tend to elicit eyeblink CR that peak earlier than higher tones. ***J***, There was no effect of sound frequency on the latency to CR onset. For complete statistics, we refer to Tables 2-Tables 3.

After 10 d of acquisition training, we tested the stimulus generalization for seven consecutive days. During these generalization test sessions mice were subjected to GSs, with frequencies ranging from 2 to 8 and 12 to 20 kHz. Importantly, these GS were never reinforced with the air puff US. The GSs had the exact same duration of 280 ms and ramp/decay times of 25 ms as the 10-kHz CS. Since the generalization test sessions consisted of substantially more trials wherein the tone was not reinforced with an air puff US (see Materials and Methods), we carefully checked whether this would lead to any extinction of learned eyeblink CRs. We found no significant effect of session on CR percentage (Fig. 5*A*; *F*_(6,60)_ = 0.60; *p* = 0.73, ANOVA on LME) and thus concluded that there was no extinction of eyeblink CRs over the course of seven generalization test sessions. This allowed us to pool the data of all seven generalization test sessions to study the effect of GS tone frequency on CR percentage, CR amplitude, and CR timing using the exact same criteria that were used for analyzing the acquisition training data.

**Table 4:** *Post hoc* comparison between the 10-kHz CS and all other GS frequencies for cumulative CR amplitude calculated over all trials and cumulative CR amplitude calculated over CR only trials

CS 10-kHz vs GS	2 kHz	4 kHz	6 kHz	8 kHz	12 kHz	14 kHz	16 kHz	18 kHz	20 kHz
CR amp-all trials	*p* < 0.0001	*p* < 0.0001	*p* < 0.0001	*p* = 0.0110	*p* = 1.092	*p* = 0.0105	*p* < 0.0001	*p* < 0.0001	*p* < 0.0001
CR amp-CR only trials	*p* < 0.0001	*p* = 0.0032	*p* = 0.0562	*p* = 0.0095	*p* = 0.621	*p* = 0.1067	*p* < 0.0001	*p* < 0.0001	*p* < 0.0001

All values represent FDR corrected *post hoc* comparisons using a two-sample Kolmogorov–Smirnov test on the cumulative distribution. CS, conditional stimulus; GS, generalization stimulus.

#### CR percentage

**Figure 6.**
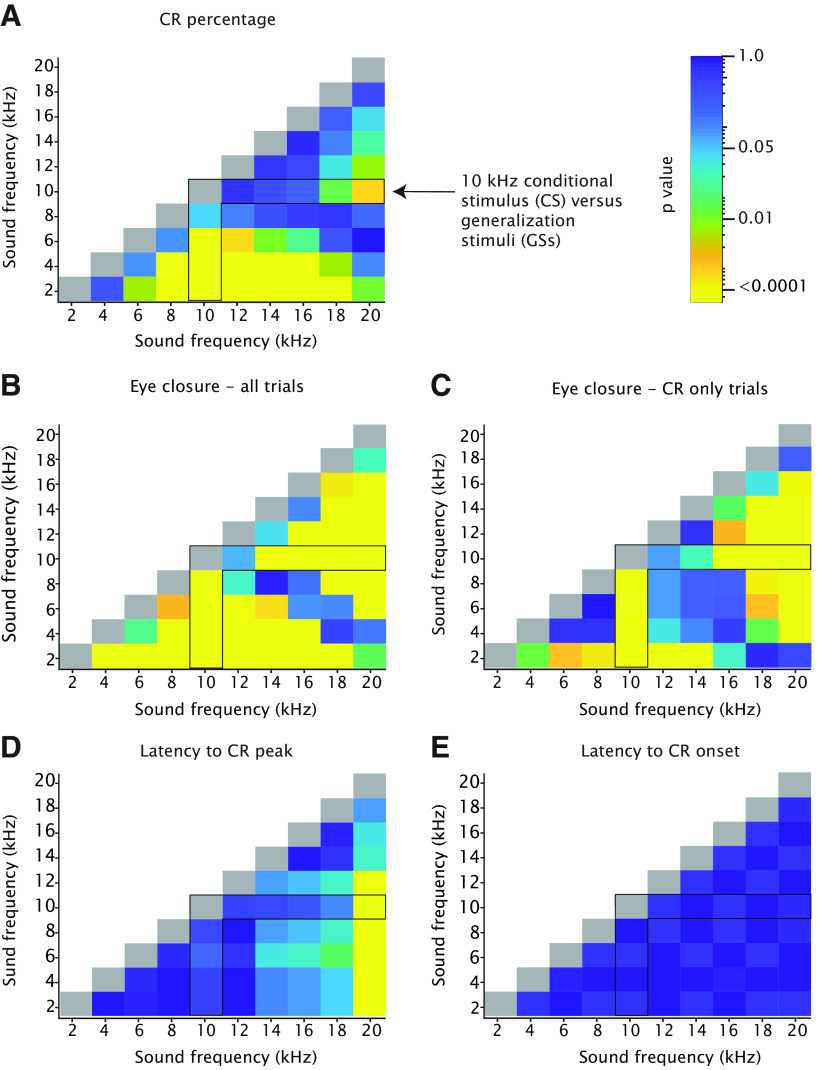
Heatmaps showing adjusted *p*-values of all tone-tone comparisons for CR percentage, CR amplitude, and CR timing. ***A***, Effect of tone frequency on CR percentage. The angular forked black box highlights the comparison between the 10-kHz CS and all the GSs. Note that the heatmap is on a logarithmic scale. All *p*-values were adjusted for multiple comparisons using FDR. Values correspond with those of Figure 5*B*. ***B***, Effect of tone frequency on eyelid closure calculated over all trials. Values correspond with those of Figure 5*C*. ***C***, Effect of tone frequency on eyelid closure calculated over CR only trials using a 0.05 criterium. Values correspond with those of Figure 5*F*. ***D***, Effect of tone frequency on latency to CR peak. Values correspond with those of Figure 5*I*. ***E***, Effect of tone frequency on latency to CR onset. Values correspond with those of Figure 5*J*. For complete statistics, we refer to Tables 2, 3.

We found a significant main effect of tone frequency on CR percentage (Figs. 5*B*, 6*A*; Table 2; *F*_(9,726)_ = 11.99, *p* < 0.0001, ANOVA on LME) with a downward gradient for CR percentage in both directions, i.e., in the direction of frequencies higher *and* lower than the 10-kHz CS tone, although this gradient appeared less pronounced for the higher frequencies. For the 10-kHz tone, mice had a CR percentage of 67 (±5), whereas for 2 and 20 kHz, we found percentages of 38 (±6) and 55 (±6), respectively (all values: mean ± 95% CI). *Post hoc* comparison revealed that GSs with frequencies of 12–16 kHz did not result in significantly different CR percentages compared with the CS, whereas GSs with frequencies between 2 and 8 and 18 and 20 kHz were statistically significant (Fig. 6*A*). We did not find an effect of sex on CR probability, male and female mice showed identical generalization curves (Fig. 9*B*; *F*_(9,1)_ = 0.45, *p* = 0.51, ANOVA on LME). We conclude that the CR probability decreased as the GSs were more different from the trained CS and that this effect was stronger for lower frequencies than for higher frequencies.

**Table 5:** Overview of previous studies on stimulus generalization and Pavlovian eyeblink conditioning studies

Authors	Animals	CS type	US	CR	Methods	Analysis	Results	Training type	CS	Probe CSs
Siegel et al. (1968)	Rabbit	Tone	4-mA shock	NM response	DC signal	(i)CR percentage single subject/average	CR decremental gradient	Nondifferential	0.5 kHz 75 dB	0.5, 1.2, 2.0, 3.0, 4.0 kHz dB
		Tone							1.2 kHz 75 dB	0.5, 1.2, 2.0, 3.0, 4.0 kHz dB
		Tone							2.0 kHz 75 dB	0.5, 1.2, 2.0, 3.0, 4.0 kHz dB
		Tone							3.0 kHz 75 dB	0.5, 1.2, 2.0, 3.0, 4.0 kHz dB
		Tone							4.0 kHz 75 dB	0.5, 1.2, 2.0, 3.0, 4.0 kHz dB
Hupka et al. (1969)	Rabbit	Tone	2-mA shock	NM response	DC signal	(i) CR percentage average	CR decremental gradient steeper for the CS+ 0.4 kHz	Differential	CS+ 0.4 kHz CS–1.6 kHz	0.4, 0.8, 1.6, 2.2, 2.8, 3.4, 4.0 kHz
		Tone						Differential	CS+ 1.6 kHz CS– 2.8 kHz	0.4, 0.8, 1.6, 2.2, 2.8, 3.4, 4.0 kHz
		Tone						Differential	CS+ 2.8 kHz CS– 4.0 kHz	0.4, 0.8, 1.6, 2.2, 2.8, 3.4, 4.0 kHz
		Tone						Differential	CS+ 1.6 kHz CS– 0.4 kHz	0.4, 0.8, 1.6, 2.2, 2.8, 3.4, 4.0 kHz
		Tone						Differential	CS+ 2.8 kHz CS– 1.6 kHz	0.4, 0.8, 1.6, 2.2, 2.8, 3.4, 4.0 kHz
		Tone						Differential	CS+ 4.0 kHz CS– 2.8 kHz	0.4, 0.8, 1.6, 2.2, 2.8, 3.4, 4.0 kHz
Swadlow and Schneiderman (1970)	Rabbit	Electrical stimulat	5-mA shock	Eyelid response		(i)CR percentage average	CR decremental gradient for frequency + duration/intensity, but no frequency + TSE	Nondifferential	LGN 1.5 s trains (21 pps, 0.21 ms)	LGN-a 1.5 pulse trains (18 test param with change in frequency + TSE/pulse dur/ intensity)
Liu (1971)	Rabbit	Tone	2-mA shock	NM response	EMG	(i)CR percentage average	CR decremental gradient peak at probe CS (1.4 kHz 75 dB)	Nondifferential	CS+ 2.4 kHz 75 dB CS+ 1.2 kHz 75 dB (C2*)	0.4, 0.8, 1.2, 1.6, 2.0 kHz 75 dB
		Tone					CR decremental gradient steeper at CS+ in T-T and T-L than C1	Nondifferential	1.2 kHz 75 dB (C1*)	0.4, 0.8, 1.2, 1.6, 2.0 kHz 75 dB
		Tone						Differential	CS+ 1.2 kHz 75 dB CS– 2.4 kHz 75 dB (T-T*)	0.4, 0.8, 1.2, 1.6, 2.0 kHz 75 dB
		Tone/light						Differential	CS+ 1.2 kHz 75 dB CS– light (T-L*)	0.4, 0.8, 1.2, 1.6, 2.0 kHz 75 dB
		Tone					CR decremental gradient slope steeper along (F) than (I)	Differential	CS+ 1.2 kHz 75 dB CS– 2.4 kHz 60 dB (I + F*)	0.4, 0.8, 1.2, 1.6, 2.0 kHz 75 dB 1.2 kHz 65, 70, 80, 85 dB
Moore (1972)	Rabbit	Tone	2-mA shock	Eyelid response	EMG	(i)CR percentage average	CR decremental steeper in T-T and T-L	Nondifferential	1.2 kHz (T)	0.4, 0.8, 1.2, 1.6, 2.0 kHz
		Tone						Differential	CS+ 1.2 kHz CS– 2.4 kHz (T-T)	0.4, 0.8, 1.2, 1.6, 2.0 kHz
		Tone/light						Differential	CS+ 1.2 kHz CS– light (T-L)	0.4, 0.8, 1.2, 1.6, 2.0 kHz
Moore and Mis (1973)	Rabbit	Tone	2-mA shock	NM response	DC signal	(i)CR percentage average	CR percentage to CS-lower for differential training	Nondifferential	0.9 kHz 75 dB	0.3, 0.6, 0.9, 1.2, 1.5, 1.8, 2.1 kHz 75 dB
		Tone						Nondifferential	0.9 kHz 95 dB	0.3, 0.6, 0.9, 1.2, 1.5, 1.8, 2.1 kHz 75 dB
		Tone						Nondifferential	1.5 kHz 75 dB	0.3, 0.6, 0.9, 1.2, 1.5, 1.8, 2.1 kHz 75 dB
		Tone						Nondifferential	1.5 kHz 95 dB	0.3, 0.6, 0.9, 1.2, 1.5, 1.8, 2.1 kHz 95 dB
		Tone						Differential	CS+ 1.5 kHz 75 dB CS– 0.9 kHz 75 dB	0.3, 0.6, 0.9, 1.2, 1.5, 1.8, 2.1 kHz 75 dB
		Tone						Differential	CS– 0.9 kHz 75 dB CS– 1.5 kHz 75 dB	0.3, 0.6, 0.9, 1.2, 1.5, 1.8, 2.1 kHz 75 dB
		Tone						Differential	CS+ 1.5 kHz 75 dB CS– 0.9 kHz 95 dB	0.3, 0.6, 0.9, 1.2, 1.5, 1.8, 2.1 kHz 95 dB
		Tone						Differential	CS+ 1.5 kHz 95 dB CS– 0.9 kHz 75 dB	0.3, 0.6, 0.9, 1.2, 1.5, 1.8, 2.1 kHz 95 dB
Solomon and Moore (1975)	Rabbit	Tone	2-mA shock	NM response	DC signal	(i)CR percentage average	(i) CR percentage gradient less steep for HP and CTX group	Nondifferential	1.2 kHz 76 dB	0.4, 0.8, 1.2, 1.6, 2.0 kHz 76 dB
		Tone						Nondifferential	1.2 kHz 76 dB *(lesion HP)*	0.4, 0.8, 1.2, 1.6, 2.0 kHz 76 dB
		Tone						Nondifferential	1.2 kHz 76 dB *(lesion CTX)*	0.4, 0.8, 1.2, 1.6, 2.0 kHz 76 dB
Kehoe et al. (1995)	Rabbit	Tone	2-mA shock	NM response	Photoelectric transducer	(i)CR percentage/amplitude/onset average	(i)CR onset earlier in (A), (D) (ii)CR amplitude/percentage lower in (A) and (D) than (S)	Nondifferential		0.5 kHz 55 dB 0.5–1.5(A*), 1.5–0.5(D*), 0.5 kHz(S*)
		Tone						Nondifferential	1.0 kHz 55 dB	0.5–1.5, 1.5–0.5, 1.0 kHz
		Tone						Nondifferential	1.5 kHz 55 dB	0.5–1.5, 1.5–0.5, 1.0 kHz
		Tone					(i)CR onset earlier in (A), (D)	Nondifferential	60 dB	60–90 dB, 90–60 dB, 60 dB
		Tone						Nondifferential	75 dB	60–90 dB, 90–60 dB, 75 dB
		Tone						Nondifferential	90 dB	60–90 dB, 90–60 dB, 90 dB
		Tone					(i)CR decremental gradient likelihood/amplitude (ii) CR onset unchanged	Nondifferential	50 ms	50, 400, 800, 1600 ms
		Tone						Nondifferential	400 ms	50, 400, 800, 1600 ms
		Tone						Nondifferential	800 ms	50, 400, 800, 1600 ms
Svensson et al. (1997)	Ferrets	electric stimulat	3-mA shock	Eyelid response	EMG	(i)CR onset/peaktime average	(i)CR onset/peaktime earlier	Nondifferential	Left FL* 300 ms 50 Hz 1 mA	Left FL 300 ms 50 Hz 2 mA
		electric stimulat						Nondifferential	MCP* 0.1 ms 50 Hz	MCP 0.1 ms 100 Hz
Garcia et al. (2003)	Rabbit	Tone	4-mA shock	(i)NM response (ii) eyelid response	(i) infrared LED (ii) photoelectric transucer	(i)CR percentage/amplitude/onset/peaktime average	(i)CR percentage/amplitude decremental gradient (ii)CR onset/peaktime increment	Nondifferential	1.0 kHz 75 dB	1.0, 1.26, 1.59, 2.0, 2.52, 3.17, 4.0, 5.04 kHz 75 dB
Ohyama et al. (2003)	Rabbit	Tone	0.8- to 2.5-mA shock	Eyelid response	Infrared LED	(i)CR/SLR percentage/amplitude average	(i)SLR/CR decremental gradient (excl light) (ii)CR amplitude unchanged	Nondifferential	1.0 kHz 85 dB	1.0, 1.85, 3.55, 6.1, 9.5 85 dB, light
		Tone					*(I)SLR/CR amplitude decremental gradient*	*Nondifferential*	1.0 kHz 85 dB (PCX*) cerebellar cortex	*1.0, 1.85, 3.55, 6.1, 9.5 85 dB, light*
		Tone					(i)SLR/CR decremental gradient (excl light) (ii)CR amplitude unchanged	Nondifferential	9.5 kHz 85 dB	1.0, 1.85, 3.55, 6.1, 9.5 85 dB, light
		Tone					*(I)SLR/CR amplitude decremental gradient*	*Nondifferential*	*1.0 kHz 85 dB (PCX) cerebellar cortex*	*1.0, 1.85, 3.55, 6.1, 9.5 85 dB, light*
		light					(i)SLR/CR light only	Nondifferential	Light	1.0, 1.85, 3.55, 6.1, 9.5 85 dB, light
Svensson et al. (2010)	Ferrets	Electrical stimulat	3-mA shock	Eyelid response	EMG	(i) SS suppression single PCs	(i) SS* suppression earlier in PCs (ii) PCs fire freq unchanged	Nondifferential	FL 0.5 kHz 300 ms 1 mA pulse train	FL 0.5 kHz 300 ms 2 mA pulse train
		Electrical stimulat	CF 0.50 kHz 10 ms (x2)	Eyelid response	EMG	(i) SS suppression single PCs	(i)SS suppression earlier in PCs	Nondifferential	MF* 0.5 kHz 400–800 ms pulse train	MF 1.0 kHz 400–800 ms pulse train
Khilkevich et al. (2018)	Rabbit	Tone	1- to 3-mA shock	Eyelid response	Infrared LED	(i)CR percentage/amplitude average	(i) CR decremental gradient (ii) CR amplitude unchanged	Nondifferential	1.0 kHz 75 dB 500 ms	1.0 kHz 75 dB 50, 100, 150, 200, 250, 300, 350, 400, 450 ms
		Electrical stimulat						Nondifferential	MF pulse train 100 Hz 100–150 mA	MF pulse train 90, 80, 70, 60, 50 Hz 100–150 mA
		Electrical stimulat						Nondifferential	MF pulse train 100 Hz 100–150 mA	MF (competing) pulse train 100 Hz 100–150 mA

Note that none of these studies was done in mice.

C1, 1 CS (tone/light); C2, 2 CSs (reinforced tone); T, 1 CS tone; T-T, tone-tone; T-L, tone-light; F + I, frequency + intensity; A, ascending tone; D, descending tone; S, steady tone; FL, forelimb; MCP, middle cerebellar peduncle; MF, mossy fibers; PCX, picrotoxin; TSE, total stimulus energy; LGN,lateral geniculate nucleus; HP, hippocampus; CTX, cortex

#### CR amplitude, all trials

**Figure 7.**
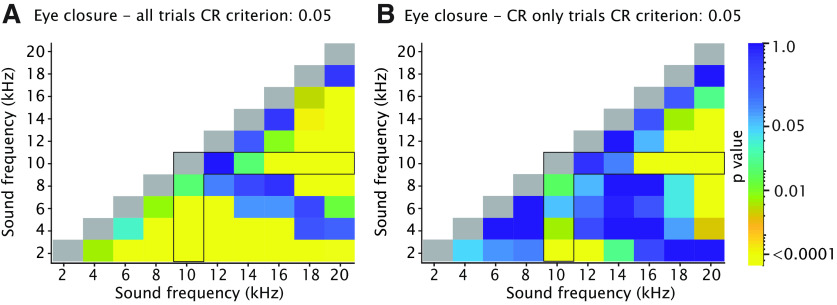
Heatmaps showing adjusted *p*-values of all tone-tone comparisons for cumulative CR amplitude. ***A***, Effect of tone frequency on cumulative CR amplitude calculated over all trials. Color indicates *p*-value. The angular forked black box highlights the comparison between the 10-kHz CS and all GSs. Note that the heatmap is on a logarithmic scale. All *p*-values were calculated using a Kolmogorov–Smirnov test on the cumulative distribution function (CDF). All *p*-values were adjusted for multiple comparisons using FDR. Values correspond with those of Figure 5*E*. ***B***, Similar to ***A*** but now for the effect of tone frequency on cumulative CR amplitude calculated over CR only trials using 0.05 criterium. Values correspond with those of Figure 5*H*. For complete statistics, we refer to Table 4.

When looking at all CS-only trials, we found a significant main effect of tone frequency (Fig. 5*C*,*D*; Table 2; *F*_(9,4849)_ = 44.34, *p* < 0.0001, ANOVA on LME), with a clear downward gradient in both directions, i.e., in the direction of frequencies higher and frequencies lower than the 10-kHz CS tone. Compared with CR percentage, the curves for both the CR amplitude calculated over all trials looked more symmetric. For the 10-kHz tone, mice had a CR amplitude calculated over all trials of 0.51 (±0.04), whereas for 2 and 20 kHz they had amplitudes of 0.20 (±0.04) and 0.29 (±0.04), respectively (all values: mean ± 95% CI). *Post hoc* comparison revealed that GSs with frequencies close to the 10-kHz CS (12 kHz) did not result in significantly different CR amplitudes calculated over all trials, whereas GSs with frequencies equal or higher than 14 kHz or equal or lower than the 8-kHz CS were all significantly different (Fig. 6*B*; Table 3). When comparing the cumulative distributions of CR amplitudes calculated over all trials we found significant effects for all GS frequencies, except for the 12-kHz GS (Figs. 5*E*, 7*A*; for *p*-values, we refer to Table 4; all Kolmogorov–Smirnov test with correction for multiple comparison using FDR).

#### CR amplitude, CR only trials

**Figure 8.**
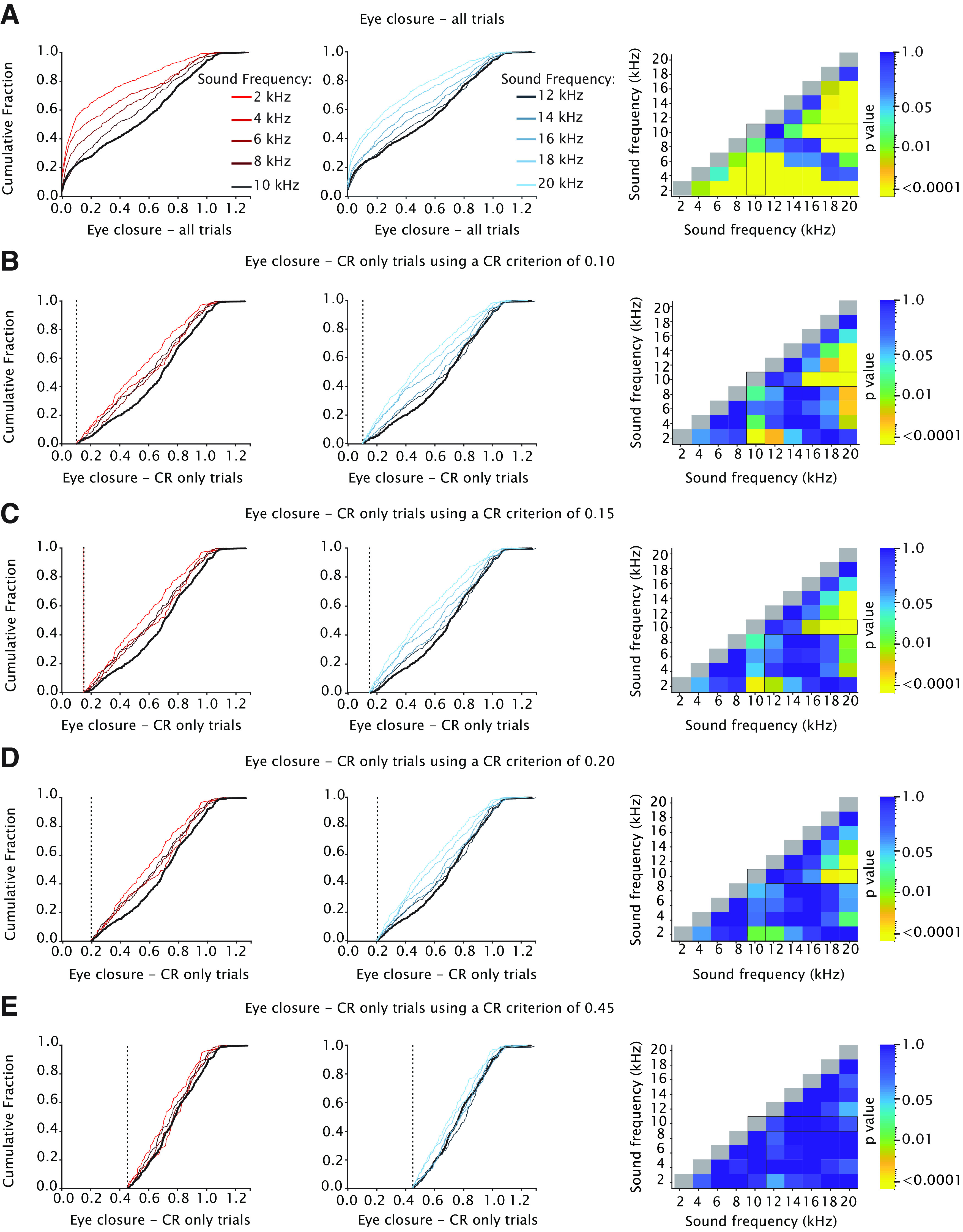
Heatmaps showing adjusted *p*-values of all tone-tone comparisons for cumulative CR amplitude using different CR thresholds. ***A***, Effect of sound frequency on cumulative CR amplitude calculated over all trials. Color indicates *p*-value. The angular forked black box highlights the comparison between the 10-kHz CS and all GSs. Note that the heatmap is on a logarithmic scale. All *p*-values were calculated using a Kolmogorov–Smirnov test on the cumulative distribution function (CDF). All *p*-values were adjusted for multiple comparisons using FDR. Similar to Figure 5, the blue gradient indicates GSs with frequencies higher than the 10-kHz CS and red gradient indicates GSs with frequencies lower than the 10-kHz CS. ***B–D***, Similar to ***A*** but now using a CR criteriums of 0.10, 0.15, and 0.20 FEC. ***E***, Similar to ***B–D*** but now using the lowest CR threshold whereby there is a nonsignificant effect of GS for all frequencies. In mice, this threshold appeared to be 0.45. Thus, a threshold of 0.45 FEC was needed to get a binary response pattern, as reported previously (Khilkevich et al., 2018), whereby the probability of a CR gradually decreases depending on the similarity between CS and GS, but the amplitude of the CR remained constant. Note that this 0.45 is close to the split of the bimodal distributions shown in Figure 4*D*,*F*.

We found a significant main effect of tone frequency for CR amplitude calculated over only trials with a CR (Fig. 5*F*,*G*; Table 2; *F*_(9,2692)_ = 16.70, *p* < 0.0001, ANOVA on LME), with a downward gradient in both directions, i.e., in the direction of frequencies higher and frequencies lower than the 10-kHz CS tone. For the 10-kHz tone, mice had a CR amplitude calculated over CR trials of 0.63 (±0.04), whereas for 2 and 20 kHz, we found amplitudes of 0.42 (±0.05) and 0.46 (±0.05), respectively (all values mean ± 95% CI). Similar to CR amplitude calculated over all CS trials, we found that *post hoc* comparison revealed that GSs with frequencies close to the 10-kHz CS (12 kHz) did not result in significantly different CR amplitudes whereas GSs with frequencies equal or higher than 14 kHz or equal or lower than the 8-kHz CS were all significantly different (Fig. 6*C*; Table 3). Interestingly, when comparing the cumulative distributions of CR amplitudes calculated over CR only trials, we found a pattern that looked slightly different from the one we found for CR amplitude calculated over all trials (Fig. 5*H*). Although there was still a clear gradient, the range was narrower and GS frequencies of 6, 12, and 14 kHz did not result in statistically significant CR amplitudes (Fig. 7*B*; for *p*-values we refer to Table 4. All Kolmogorov–Smirnov test with correction for multiple comparisons using FDR). Previous work in rabbits showed a “binary choice phenomenon” (Khilkevich et al., 2018) whereby the probability of CRs gradually decreased on the degree of similarity between the GS and CS, but the amplitude of the CR remained constant. Since the CR threshold of 0.05 FEC is rather arbitrary, we also looked at higher CR thresholds of 0.10, 0.15, 0.20, and 0.25 but could not establish a binary choice phenomenon (Fig. 8*A–D*). Lastly, we established the threshold that would provide us with a binary choice, by step-wise increasing the CR threshold. We found that a CR threshold of 0.45 was needed to get a nonsignificant effect of any of the tone frequencies (Fig. 8*E*).

**Figure 9.**
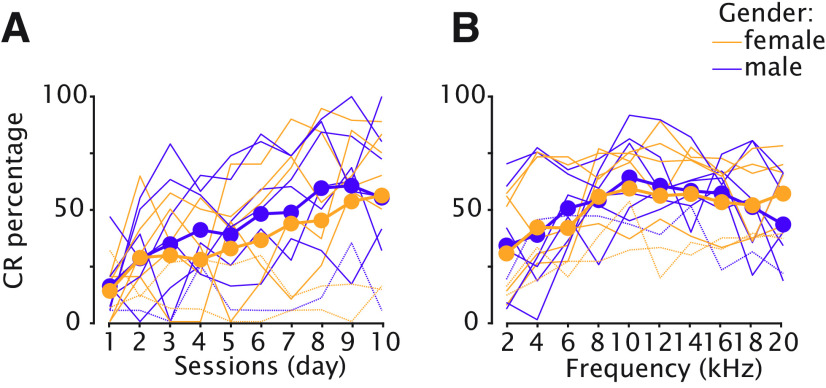
No effect of sex on acquisition and generalization of conditioned eyeblink responses. ***A***, CR percentage during acquisition sessions 1–10. Each colored line represents an individual mouse (yellow for females, purple for males). All animals are included (*n* = 14), also the ones that did not learn the task (*n* = 3) and are therefore excluded from the main statistical analysis of this paper. Thicker lines indicate the averages respectively for males and females including all the animals. ***B***, Generalization test sessions pooled together. Same color coding as in ***A***. Here again, all animals are included in the averages.

#### Latency to CR onset and peak

Finally, we studied the effect of GS frequency on the timing of eyeblink CRs. As measures for CR timing, we looked at latency to CR onset and latency to CR peak. We found a significant main effect of tone frequency on latency to CR peak (Figs. 5*I*, 6*D*; Table 2; *F*_(9,2692)_ = 5.56, *p* < 0.0001, ANOVA on LME). Interestingly, we found a gradient whereby it appeared that the lowest frequencies resulted in CR peaks with the shortest latencies and the highest frequencies in CR peaks with the longest latencies. *Post hoc* comparison revealed that only the GS with the highest frequency (20 kHz) resulted in a significantly longer latency to CR peak compared with those to the 10-kHz CS. We thus conclude that tone frequency in mice has no effect on the latency to CR onset, but does have a mild effect on latency to CR peak. We found no significant main effect of tone frequency on latency to CR onset (Figs. 5*J*, 6*E*; Table 2; *F*_(9,322)_ = 1.12, *p* = 0.34, ANOVA on LME). Regardless of CS or GS tone frequency, the latency to CR onset was around 150 ms after CS onset.

## Discussion

We found that mice show a strong generalization of CRs in Pavlovian eyeblink conditioning using a tone as CS. Both CR probability and CR amplitude decreased as the GSs were more different from the CS. We also found a minor effect on the adaptive timing of eyeblink CRs whereby the tone lowest frequencies resulted in CR peaks with the shortest latencies and the highest frequencies in CR peaks with the longest latencies. No effect was found for latency to CR onset. Hence, our study provides a first investigation of stimulus generalization for eyeblink conditioning in mice using GSs with tone frequencies higher and lower than the CS.

The cerebellum embodies more than two-thirds of all neurons in our brain and takes part to a large extent in the formation of procedural memories in motor behavior (De Zeeuw and Ten Brinke, 2015). Converging evidence highlights the role for cerebellum also in nonmotor functions, such as social cognition (Van Overwalle et al., 2015), emotional processing (Schmahmann and Caplan, 2006), social behavior (Schmahmann and Sherman, 1998), addiction (Volkow et al., 2003; Miquel et al., 2009; Moulton et al., 2014), and fear learning (Maschke et al., 2003; Lange et al., 2015). Based on our finding that mice show a strong stimulus generalization in eyeblink conditioning, we expect that cerebellum is capable to regulate stimulus generalization both in motor and nonmotor domains.

### Differential versus nondifferential training

Eyeblink conditioning can be performed according to a nondifferential or differential protocol. During nondifferential training, which is the paradigm we employed in our current study, subjects are trained with only one CS, for instance a 10-kHz tone, and tested with GSs after acquisition training is finished. During differential training, instead, subjects are trained with more than one CS, whereby one CS (CS+) is reinforced with a US and one or several other CSs are not reinforced at all (CS-). Since previous work has shown that these different eyeblink conditioning protocols have an effect on the stimulus generalization gradient (Hupka et al., 1969; Moore and Mis, 1973), we will mainly compare our findings with previous studies using a nondifferential protocol.

### CR percentage

CR probability decreases with each incremental or decremental 2-kHz step away from the trained 10-kHz CS. Still, mice show CRs in a respectable amount of the trials at the upper and lower limits that we tested in this study: where the 10-kHz CS evoked CRs in ∼67% of the trials, the lower limit 2-kHz and upper limit 20-kHz probe CS evoked CRs in 38% and 55% of the trials, respectively. This gradient in CR probability was seen both at a mouse individual level as well as a group level (Fig. 5*B*), indicating that the gradient was not simply a smoothing effect because of averaging data (Razran, 1949). Previous studies on stimulus generalization during nondifferential eyeblink conditioning primarily looked at CR percentage (i.e., CR probability; Table 5). Rabbits generally show a CR probability pattern that looks very similar to the one we observed in mice: the highest CR probability to the trained CS and progressive decrease in response probability to more distant GS frequencies (Moore, 1964; Siegel et al., 1968; Moore and Mis, 1973; Solomon and Moore, 1975; Garcia et al., 2003; Khilkevich et al., 2018). None of these studies, however, assessed GSs in both directions of the frequency spectrum, i.e., for tone frequencies higher and lower than the CS (Table 5). Interestingly, rabbits trained using a differential eyeblink conditioning protocol yielded a steeper CR gradient in stimulus generalization testing than those trained in a nondifferential procedure (Moore, 1964; Liu, 1971).

### CR amplitude

Similar to CR percentage, the amplitude of conditioned eyelid closure calculated across all CS trials shows a stepwise decrease when the difference between the trained CS and GS increases. Since CR probability and amplitude of eyelid closure show a strong covariation on a single session level, this is not a surprising result. Indeed, previous studies done in rabbits, show the same phenomenon for eyelid closure calculated over all trials. For instance, Garcia et al. (2003) and Ohyama et al. (2003) report that the magnitude (i.e., CR amplitude calculated over all CS trials) of eyeblink responses shows a progressive decrease with each incremental step in tone frequency away from the CS. In addition, Khilkevich et al. (2018), using electrical stimulation of mossy fibers as CS, similarly show that GSs with stimulation frequencies lower than the CS result in a lower CR amplitude.

Only a subset of previous studies also describes the amplitude of eyelid closure for only those trials wherein the animal shows a CR (Kehoe et al., 1995; Garcia et al., 2003; Khilkevich et al., 2018). Looking at this value in our study, using a 0.05 CR criterion, we again observed the same gradient with the highest CR amplitude to the trained CS and a progressive decrease in CR amplitude for each incremental or decremental 2-kHz step away from the CS. Although this gradient was less steep than for CR amplitude calculated over all trials, we could not establish the clear binary choice phenomenon reported by Khilkevich et al. (2018), whereby the probability of CRs gradually decreased on the degree of similarity between the GS and CS, but the amplitude of the CR remained constant. Since a FEC of 0.05 is an arbitrary CR threshold, we also looked at higher CR thresholds (Fig. 8*B–E*) but could not establish the binary choice. The most parsimonious explanation for this discrepancy between our study and Khilkevich et al. (2018) is the difference in the eyelid motor plant between mice and rabbits. In mice, the main force driving eyeblink CRs comes from contraction of the orbicularis oculi muscle, while in rabbits (and humans) there is, in addition to the contraction of the orbicularis oculi muscle, a more pronounced role for a simultaneous relaxation of the levator palpebrae muscle (Ansari and Nadeem, 2016). This results in a different CR expression profiles, whereby conditioned rabbits show a clear bimodal (or better: zero-inflated) distribution of CR amplitudes (Garcia et al., 2003; Khilkevich et al., 2018). For mice, this bimodal distribution is present, but clearly less obvious compared with the ones reported for rabbits (Kloth et al., 2015; ten Brinke et al., 2017; Albergaria et al., 2018; compare histograms in Fig. 4*D*,*F* with those reported by Khilkevich et al., 2018, their Fig. 2*A*).

Another difference between our study and the Khilkevich et al. (2018) study is the performance level of the animals at the end of training. Khilkevich et al. (2018) trained their rabbits “until both CR percentage was high (CR% > 90%) and CR amplitudes were robust and near the target amplitude.” In practice, this meant that most rabbits were trained for 10 sessions. In our experiment, mice reached maximum conditioning levels of ∼70% CRs and CR amplitudes of ∼0.5. These values for our mice did not further increase and remained stable after acquisition session 8. Thus, the Khilkevich et al. (2018) rabbits were clearly performing better than our mice: the rabbits were overtrained, whereas in our mice there was theoretically still room for further improvement. This difference in performance level could also explain why Khilkevich et al. (2018) report a binary choice and we observe more a continuum of responses.

### CR timing

Most previous studies on stimulus generalization during eyeblink conditioning ignored the adaptive timing properties of eyelid CRs (Moore, 1964; Siegel et al., 1968; Liu, 1971; Moore and Mis, 1973; Khilkevich et al., 2018). Our data show that mice CRs peaked significantly later to GSs with higher frequencies compared with those with lower frequencies. These findings are in line with response patterns described by Garcia et al. (2003). Interestingly, when electrical stimulation of the forelimb, that had served as a CS, was suddenly switched from 50 kHz to a 100-kHz stimulus train, an opposite effect was reported: the latency to CR peak was shorter for the higher frequency stimulus (Svensson et al., 1997). We have no clear explanation for this effect of tone frequency on latency to CR peak. It may reflect processing of auditory information between the level of sensory organs (cochlea) and the cerebellar mossy fiber input system.

In mice, there is no effect of tone frequency on the latency to CR onset. This finding is in line with previous work in mice, showing that the latency to CR onset is rather unaffected by the duration of the CS (Chettih et al., 2011), which is another difference between eyeblink CRs in mice compared with other species (rabbits, humans, ferrets). Indeed, a trending (but not significant) increase in CR onset latency has been described for stimulus generalization in rabbits (Garcia et al., 2003).

### Latent inhibition

Three out of fourteen animals did not learn the task within the 10 d of acquisition training, which is slightly higher compared with previous work by our group (Boele et al., 2018; Grasselli et al., 2020; Beekhof et al., 2021; de Oude et al., 2021). The difference between this study and previous work, is the amount of CS preexposure during the 10 baseline sessions, which potentially leads to “latent inhibition.” Latent inhibition is the phenomenon whereby it takes longer to get conditioned to a familiar stimulus than to novel stimulus. The preexposure to the CS (20 in total) and GS (20 in total for each frequency) during the 10 baseline sessions could explain why these three animals did not learn the task. Although we made sure all animals received the exact same amount of CS-only and GS-only trials during the baseline sessions to keep the novelty level of the tone equal for all animals, it could be that the latent inhibition effect varies between animals.

### Comparison between generalization curves from eyeblink conditioning and fear conditioning

Stimulus generalization has been studied previously using fear conditioning. However, to our knowledge, a complete assessment of conditioned fear responses as a function of a wide range of tone frequency is missing: all studies probed generalization of fear responses presenting only one or a few novel auditory cue(s) (Shaban et al., 2006; Zhang et al., 2019). In addition, most fear conditioning studies use a differential paradigm during acquisition. For these two reasons combined, it is almost impossible to compare our eyeblink conditioning generalization curve with those using fear conditioning. It would be interesting to find out how US intensity affects the shape of the generalization curve. One could do so using eyeblink conditioning, using a stronger US, which is known to induce more fear (Boele et al., 2010) and leads to faster acquisition (Passey, 1948; Spence et al., 1953; Smith, 1968; Freeman et al., 1993; Kehoe and White, 2002; see Boele et al., 2016). Based on work done on fear conditioning (Laxmi et al., 2003; Dunsmoor et al., 2009, 2017), we predict that a more aversive US leads to stronger generalization (i.e., a less steep gradient). In addition, the training paradigm (differential vs nondifferential training) has effects on the shape of the curve (Dunsmoor and LaBar, 2013).

### Neural mechanisms

The study of stimulus generalization primarily comes from fields of ethology or experimental psychology and has been investigated with various experimental paradigms other than Pavlovian eyeblink conditioning, such as fear conditioning and operant conditioning. These investigations on stimulus generalization have been performed in many species including humans, goldfishes, rats and pigeons, and generally report a decreasing generalization gradient when moving away from the trained stimulus (Thomas and Mitchell, 1962; Baron, 1973; Ghirlanda and Enquist, 2003), similar to what we found in Pavlovian eyeblink conditioning in mice. Interestingly, Guttman and Kalish (1956) showed that stimulus generalization does not originate from a failure in perceptual discrimination, but instead it is an active process. This principle probably also applies to cerebellar learning rules during eyeblink conditioning. Although early reports on stimulus generalization in eyeblink conditioning have shown that lesions of noncerebellar structures, for instance hippocampus or cerebral cortex, affect eyeblink conditioning and stimulus generalization in eyeblink conditioning (Solomon and Moore, 1975), the leading idea now is that the essential eyeblink conditioning memory trace is formed in cerebellum (McCormick et al., 1981, 1982; Mauk and Donegan, 1997; Yeo and Hesslow, 1998; Mauk and Buonomano, 2004; Freeman and Steinmetz, 2011; Heiney et al., 2014; Freeman, 2015; ten Brinke et al., 2015). Purkinje cells in well-defined microzones in cerebellar cortex receive converging inputs from the mossy fiber – parallel fiber pathway, which transmits the CS, and the climbing fiber pathway, which transmits the US (De Zeeuw and Ten Brinke, 2015; De Zeeuw et al., 2021). Repeated pairing of CS and US leads to the acquisition of a simple spike pause in Purkinje cells in response to the CS (Ohmae and Medina, 2015; ten Brinke et al., 2015; Jirenhed et al., 2017; Narain et al., 2018). Although further research is needed, one could imagine that the higher and lower frequency tones are not equally represented in the parallel fiber beams and thereby contributing to the asymmetric distribution in the stimulus-response relation.

The simple spike pause in turn causes a temporary disinhibition of cerebellar nuclei neurons, which (indirectly) innervate the motor neurons that control the eyelid musculature (Halverson et al., 2015, 2018; Jirenhed et al., 2017; ten Brinke et al., 2017). In addition, mossy fiber and climbing fibers send of collaterals directly to the cerebellar nuclei. Our previous work has shown that the number of varicosities on these mossy fiber collaterals in the cerebellar nuclei increases quite robustly with eyeblink conditioning (Boele et al., 2013). Moreover, the number of these varicosities correlates positively with the amplitude of eyelid CRs, indicating that these mossy fibers are important for CR expression. Work by Ohyama and colleagues has shown that pharmaceutical disconnection of Purkinje cell inhibition from the cerebellar nuclei results in much smaller CRs to GSs, but that CRs to the trained CS remained largely the same in size, although the adaptive timing of these CRs was affected (Ohyama et al., 2003). This suggests that mossy fiber collaterals form a CS-specific pathway from the pontine nuclei to the cerebellar nuclei. We hypothesize that cerebellar cortex and nuclei play synergistic roles in CR expression and timing. GSs that resemble the CS will result in a rather similar and strong neural representation in the parallel fiber input at the Purkinje cell, resulting in a rather similar simple spike pause. GSs that are more different instead, will result in a weaker representation, leading to a weaker Purkinje cell response.

## Synthesis

Reviewing Editor: Michaël Zugaro, CNRS, Collège de France, Inserm Decisions are customarily a result of the Reviewing Editor and the peer reviewers coming together and discussing their recommendations until a consensus is reached. When revisions are invited, a fact-based synthesis statement explaining their decision and outlining what is needed to prepare a revision will be listed below. The following reviewer(s) agreed to reveal their identity: John Freeman, Zsuzsanna Callaerts-Vegh. Note: If this manuscript was transferred from JNeurosci and a decision was made to accept the manuscript without peer review, a brief statement to this effect will instead be what is listed below.

The manuscript has been evaluated by two reviewers. Here is a summary of their comments.

Stimulus generalization has been extensively studied in the context of fear conditioning as well as of clinical issues and solutions (i.e. phobias or extinction learning). Here, the authors present stimulus generalization in a different context, namely to auditory stimuli in a cerebellar learning paradigm (conditioned eyeblink).

The experiment used very precise and rigorous methods to condition mice with a 10 kHz tone followed by generalization sessions with non-reinforced presentations of lower- and higher-frequency tones (care was taken to avoid eliciting beta responses, which interfere with CR measurement in mice). The study is well executed and involves a series of important control experiments and validations, including the percentage of CRs, CR magnitude, CR amplitude, latency to peak, and onset latency. While generalization is seen across auditory frequencies, CR percentage and amplitude decrease as the test stimulus frequency differs from the trained frequency. The findings are generally consistent with the findings from rabbits, ferrets, and humans, and are very interesting from a mechanistic point of view.

The reviews are supportive, but raise a number of issues which should be fairly straightforward to address.

Major issues

The paper does not explicitly indicate why the findings are important in either the abstract or discussion. For example, fear conditioning plays an important role in many clinical pathologies, and understanding the mechanisms could potentially lead to new therapeutic approaches. The authors should make a stronger case for their protocol.

The discussion should include a section comparing generalization curves in mice trained with other conditioning procedures such as fear conditioning. The steepness of the generalization function is particularly important and might differ between eyeblink conditioning and other paradigms that use longer and/or louder tones.

Minor issues

The authors include 7 males and 7 females, but do not provide any information about the effect of sex in their study. Given the paucity of sex-balanced studies in neuroscience, it would be useful to document if there was an effect of sex or not. N=7 is for most behavioral tests lies on the low side (or n=11). Was a prospective power analysis performed to ensure that the study was not underpowered?

In the first paragraph of the Introduction, the authors cite a paper by Smith et al. 2007 as an example of an eyeblink conditioning study, even though it is a fear conditioning study.

The authors claim that their eyeblink conditioning paradigm is “in a more natural setting,” which makes little sense since the mouse is head-fixed and walking on a foam treadmill. Moreover, the use of locomotion is a confounding factor introducing variability unless one triggers trials when the mouse is in a particular state (e.g., during locomotion). Thus, the authors should simply describe the use of their paradigm as a precise way to measure conditioning, and remind the reader that it has been used in their previous studies.

In the methods section, Charisma is mentioned, but not what it is. Is it a type of glue?

For the generalization results, F statistics are presented but it is not clear if these are from ANOVAs on the LME.

In the first paragraph of the discussion the authors claim that “lower and higher frequencies resulted in CR peaks with slightly decreased and increased latencies” but there was only a significant effect for the highest frequency.

In the discussion of CR timing, it is important to note that mice differ from humans, rabbits, and rats in not showing a decrease in CR onset latency across training. This finding also differs from many other Pavlovian CRs, including salivation in Pavlov’s original experiments. Is there something about the combination of species and ISI that causes this difference?

Halverson et al. 2015 and 2018 should be cited as examples of studies showing simple spike pauses.

In several figures, the term “criterium” should be replaced with ’criteria’ or ’criterion’.

Figure S1 and Tables 1-2 may not all be needed. One possibility could be to keep Figure S1 and remove the tables.

Figure S4 is not necessary, as this correlation is well documented.
